# Sustainable pathways towards climate and biodiversity goals in the UK: the importance of managing land-use synergies and trade-offs

**DOI:** 10.1007/s11625-022-01242-8

**Published:** 2022-11-07

**Authors:** Alison C. Smith, Paula A. Harrison, Nicholas J. Leach, H. Charles J. Godfray, Jim W. Hall, Sarah M. Jones, Sarah S. Gall, Michael Obersteiner

**Affiliations:** 1 Environmental Change Institute, University of Oxford, South Parks Road, Oxford, OX1 3QY UK; 2 UK Centre for Ecology and Hydrology, Library Avenue, Bailrigg, Lancaster, LA1 4AP UK; 3 Oxford Martin School, University of Oxford, 34 Broad St, Oxford, OX1 3BD UK; 4 Lancaster University, Bailrigg, Lancaster, LA1 4YW UK

**Keywords:** Land use, Agriculture, Food, Climate change mitigation, Biodiversity conservation, Policy

## Abstract

Agricultural and environmental policies are being fundamentally reviewed and redesigned in the UK following its exit from the European Union. The UK government and the Devolved Administrations recognise that current land use is not sustainable and that there is now an unprecedented opportunity to define a better land strategy that responds fully to the interconnected challenges of climate change, biodiversity loss and sustainable development. This paper presents evidence from three pathways (current trends, sustainable medium ambition, and sustainable high ambition) to mid-century that were co-created with UK policymakers. The pathways were applied to a national integrated food and land-use model (the FABLE calculator) to explore potential synergies and trade-offs between achieving multiple sustainability targets under limited land availability and constraints to balance food supply and demand at national and global levels. Results show that under the Current Trends pathway all unprotected open natural land would be converted to urban, agriculture and afforested land, with the consequence that from 2030 onwards tree planting targets could not be met. In contrast, the two sustainable pathways illustrate how dietary change, agricultural productivity improvements and waste reduction can free up land for nature recovery and carbon sequestration. This enables a transition to a sustainable food and land-use system that provides a net carbon sink with up to 44% of land able to support biodiversity conservation. We highlight key trade-offs and synergies, which are important to consider for designing and implementing emerging national policies. These include the strong dependence of climate, food and biodiversity targets on dietary shifts, sustainable improvements in agricultural productivity, improved land-use design for protecting and restoring nature, and rapid reductions in food loss and waste.

**Supplementary Information:**

The online version contains supplementary material available at 10.1007/s11625-022-01242-8.

## Introduction

Globally, over 70% of land is directly used by humans (IPCC 2019), principally for food production. Most current food production systems are unsustainable, contributing to land degradation, climate change, loss of natural habitats and biological diversity, overuse of freshwater, and nitrogen and phosphorus pollution (Gerten et al. 2020; Rockström et al. 2009; Steffen et al. 2015). Many recent reports recognise the urgent need to transition to more sustainable land-use and agricultural practices (IPBES 2018; IPCC 2019; Sachs et al. 2019). Sustainable land use must be underpinned by biodiversity and ecosystem health, which are crucial for ensuring the long-term resilience of ecosystems to environmental change and thus the future delivery of ecosystem services (Seddon, Smith et al. 2021). This requires a holistic approach that balances trade-offs between food production and other ecosystem services (Smith et al. 2017), while also reversing biodiversity loss (Brussaard et al. 2010; Gerten et al. 2020; Lang et al. 2009; Schmidt-Traub et al. 2019).

The UK government recognises that transitioning to more sustainable land use is essential for delivering its ambitious targets on climate change and biodiversity. For climate, the UK was the first major economy to set a legally binding target to end its contribution to global warming by committing to achieve net zero greenhouse gas (GHG) emissions by 2050 (BEIS 2019). For biodiversity, the UK has committed to reversing biodiversity loss by 2030 as a signatory to the Leaders’ Pledge for Nature, and will publish a new Nature Strategy in response to the post-2020 global biodiversity targets, which are expected to be agreed in 2022. In parallel, the UK has produced a draft National Food Strategy which aims to tackle a “plague of dietary ill-health” and the “environmental damage caused by intensive agriculture” (Dimbleby 2021).

All these commitments must be delivered as the UK adapts to leaving the European Union (EU) and its Common Agricultural Policy (CAP). This will affect all aspects of the UK economy, but especially the agricultural sector, due to changes in trade and subsidies (Bateman and Balmford 2018). Agricultural and environmental policies are currently being fundamentally reviewed and redesigned by the UK Government and Devolved Administrations. New sustainable land management schemes based on payments for public goods, including nature recovery, are being designed and tested in pilots, with the aim of fully transitioning away from previous area-based payments for food production over the next few years. However, evidence to support the design of these new land-use policies is generally lacking (Thomas et al. 2021). In particular, evidence is needed to understand what actions might enable a transition to a more sustainable and healthier food and land-use system that contributes to the delivery of the UK’s climate and biodiversity targets, while supporting a viable farming sector and meeting the needs of a growing population.

Holistic models of the land-use system can provide evidence in support of the design of agricultural and environmental policies by exploring the impact of alternative pathways that incorporate actions related to trade, dietary change, afforestation, food waste and agricultural productivity on land-use change, GHG emissions and biodiversity. There are a number of existing models of food and land-use systems that could be applied to the UK. Integrated Assessment Models (IAMs) (Briassoulis 2019; Soesbergen 2016) aggregate many different sectors in a single model that can be used for scenario simulations or to optimise land use for one or more parameters, such as economic costs. However, such models often do not model biodiversity impacts and would not be easy to downscale for the UK as they typically operate using large trading blocks. Moreover, global IAMs inevitably lack insights into the cultural and political context in each of the many countries that are contained within them. This means that the scenarios and policies tested can be insensitive to local context and lack legitimacy with national stakeholders.

Existing modelling of the UK land system under alternate pathways has been limited to particular issues or ecosystem services (e.g. Cantarello et al. 2011 for carbon; Redhead et al. 2020 for biodiversity), or has been sub-national in scale (e.g. Holman et al. 2008 for two regions in England; Holman et al. 2016 for Scotland; Thomas et al. 2021 for Wales). There is one existing IAM for Great Britain (not including Northern Ireland): NEV (Natural Environment Valuation), which combines several sectoral modules (farming, wood production, greenhouse gas emissions, recreation, biodiversity) to optimise land-use outcomes for their market and social value (Bateman et al. 2014; Day et al. 2020). However, NEV and its web version NEVO (Natural Environment Valuation Online tool) for England and Wales only (NEVO 2022) do not take account of changes in demand (e.g. via dietary choices) nor international trade.

Existing models are not able to test the wide range of policy options and outcomes of interest to UK policy stakeholders, whilst considering the UK in a global context. This is important as policies are being designed not only to achieve sustainable land use and food systems within the UK, but to ensure that the UK does not export its environmental footprint to other countries. To address this gap, the international FABLE (Food, Agriculture, Biodiversity, Land use and Energy) consortium has developed a unique approach that can be tailored to model sustainable pathways of interest to UK policymakers (Jones et al. this issue; Mosnier et al., 2022). The FABLE team has developed a standardised approach to modelling national food and land-use systems in an integrated way, using either a spreadsheet model (the FABLE calculator, Mosnier et al. 2020) or a compatible spatial model. Country teams around the globe can thus parameterise and apply their own national integrated models of food and land-use systems, and these national models can then be linked together to aggregate to global impacts on climate and biodiversity while also balancing imports and exports at the global level. This allows national teams to explore the part they can play in meeting global policy ambitions for food security, climate and biodiversity whilst taking account of trade constraints, as it is not possible for all countries to attempt to meet their national targets by simply importing more food. Moreover, the FABLE analysis provides a method to establish whether national targets together add up to meet global climate, biodiversity and food security goals (Table 1), and, on the other hand, what the global impact of failure to meet national targets would be.

**Table 1:** Overview of the seven global FABLE targets and their relation to national UK targets and policy ambitions

Area	Global FABLE target	Related national targets/policy ambitions
Land and biodiversity	*A minimum share of earth’s terrestrial land supports biodiversity conservation.* No net loss by 2030 and an increase of at least 20% by 2050 in the area of land where natural processes predominate	*Leaders’ Pledge for Nature* Reverse biodiversity loss by 2030 *National Biodiversity Strategies and Action Plans (NBSAPs*) for the UK nations (England, Wales, Scotland and Northern Ireland) include mainly qualitative targets on biodiversity and deforestation with some quantitative targets, e.g. creating 500,000 ha of new wildlife habitat and increasing woodland to 12% cover in England by 2042
	*A minimum share of Earth’s terrestrial land is within protected areas.* At least 30% of global terrestrial area by 2030	
	*Zero net deforestation.* Forest gain should at least compensate for the forest loss at the global level by 2030	
Greenhouse gas emissions from Agriculture, Forestry and Other Land Use (AFOLU)	*Greenhouse gas emissions from crops and livestock* compatible with keeping the rise in average global temperatures to below 1.5 °C, which we interpret as below 4 GtCO_2_e year^−1^ by 2050 (3.9 Gt for non-CO_2_ emissions and 0.1 Gt for CO_2_ emissions)	*Net zero GHG emissions by 2050*, includes emission reduction efforts from AFOLU. Envisaged mitigation measures include increasing tree and hedgerow-planting, increased agricultural productivity and dietary change (reduced consumption of ruminant meat and dairy produce) (CCC 2018)
	*Greenhouse gas emissions and removals from Land Use, Land-Use Change, and Forestry (LULUCF*) compatible with keeping the rise in average global temperatures to below 1.5 °C. Negative global greenhouse gas emissions from LULUCF by 2050	
Food security	*Zero hunger.* Average daily energy intake per capita higher than the minimum requirement in all countries by 2030	*National Food Strategy*, includes actions targeted towards dietary ill-health and the environmental damage caused by intensive agriculture. *EatWell diet*, a healthy diet according to UK government guidelines
Freshwater	*Water use in agriculture* within the limits of internally renewable water resources, taking account of other human water uses and environmental water flows. Blue water use for irrigation < 2453 km^3^ year^−1^ (global estimates in the range of 670–4044 km^3^ year^−1^) given future possible range (61–90%) in other competing water uses	*NBSAPs include action on reforming the water abstraction regime*

In this paper, we apply the FABLE calculator to explore potential synergies and trade-offs between achieving multiple sustainability targets (for food, climate and biodiversity) within the UK, whilst also taking account of implications for global sustainability targets. We do this by applying the FABLE calculator to three pathways to mid-century (Current Trends, Sustainable Medium Ambition, and Sustainable High Ambition) that were co-created with UK policymakers.

## Method

The FABLE approach is built on extensive stakeholder engagement to ensure that the pathways tested are of interest to policymakers and are thus more likely to be implemented. This engagement takes place via a ‘Scenathon’, or scenario marathon, which is an iterative series of stakeholder consultations that progressively develops a set of pathways for testing with the FABLE calculator (see Figure S1). This paper reports the outcome for the UK of the second FABLE Scenathon exercise, which took place from autumn 2019 to spring 2020, involving 20 country teams and seven rest of the world regions (see FABLE (2020) for the global outcomes of the second FABLE Scenathon).

The aim of the Scenathon was to develop pathways to 2050 for each country that collectively deliver global climate, biodiversity and food security targets. Seven science-based global targets were agreed within the FABLE consortium based on a review of the scientific literature and consistency with international agreements (Table 1; see Mosnier et al. (2022) for further details). National targets are not defined, but can be incorporated in the national FABLE calculators to test their consistency with the global targets (see Tables 1, 2 and S1).

**Table 2:** Overview of the three UK FABLE pathways (see Table S5 for details)

Current Trends	Sustainable Medium Ambition	Sustainable High Ambition
Population: Common assumption across all pathways of medium population growth (67 million inhabitants in 2020 to 75 million in 2050)		
Imports and exports: Remains unchanged for all pathways		
Agricultural expansion: Common assumption across all pathways of no constraints on agricultural expansion		
Afforestation/reforestation		
As current: 9000 ha/year	Medium: 30,000 ha/year	High: 50,000 ha/year
Urban expansion		
As current: 26,000 ha/year	As current: 26,000 ha/year	More compact: 13,000 ha/year
Protected areas		
Stable: 27.6% of total land by 2050	Increase: 27.9% of total land by 2050	Increase: 29.6% of total land by 2050
Crop productivity		
Stable: historic yield levels	Increase: + 39% in yields for all crops	Increase: + 65% in yields for all crops
Livestock productivity		
Stable: historic levels, except for milk yield which increases by 18%, half the current rate	Stable for cattle, + 18% for milk yield, and proportional increase for poultry. No change for pork and lamb/mutton	Increase of + 27% for milk yield, other changes as Sustainable Medium Ambition Pathway
Pasture stocking rate		
As current	Increases by 10%	Increases by 50%
Post-harvest losses		
As current	Reduction of 50% by 2050	Reduction of 50% by 2030
Food waste		
As current	Reduction of 20% by 2050	Reduction of 20% by 2025
Food diets		
As current	Reduction of 20% in ruminant meat (beef and lamb) and milk consumption by 2050, replaced by increased consumption of pork, poultry, fish, eggs, pulses and nuts	Move to the EatWell diet recommendations on consumption of major food groups by 2050: a healthier and more plant-based diet, with reductions of 30% in ruminant meat, 60% in milk, 95% in pork and 80% in poultry consumption by 2050 compared to 2015 (see Table S6)
Climate change		
RCP 6.0	RCP 2.6	RCP 2.6

In this section, we first describe the FABLE calculator, and then describe how we worked with UK stakeholders to parameterise the model and define the potential pathways to sustainable land use.

### The standard FABLE calculator

The FABLE calculator is an open source national integrated land-use model implemented in Excel (Mosnier et al. 2020). It enables the rapid and transparent simulation of pathways towards sustainable land-use and food systems using national-level data, with FAOSTAT being the default source. It focuses on agriculture as the main driver of land-use change and includes 76 raw and processed agricultural products from the crop and livestock sectors. User-defined scenario assumptions are used to explore the impact of different policies and drivers on the level of agricultural activity, land-use change, food consumption, trade, GHG emissions, water use, and biodiversity conservation in 5-year time steps from 2000 to 2050.

The calculator is driven by projections of future demand for crop and livestock products based on scenario assumptions about future changes in population levels, diets, food waste and imports/exports. This demand includes tonnes of crops required for direct human consumption or for processing into intermediate products, the number of livestock needed to meet the demand for animal produce, the amount lost through food waste, and the demand for non-food uses (such as for biofuels). This is used to calculate the associated demand for cropland and pasture, taking into account demand for livestock feed crops. Scenario assumptions related to future changes in crop and livestock productivity, post-harvest crop losses and ruminant density per hectare of pasture affect the amount of land needed to satisfy this demand. Final land-use change is computed by adjusting targeted cropland and pasture to feasible cropland and pasture depending on land availability, protected areas, and competing demands for land for urban expansion and to meet user-defined afforestation targets. The final feasible crop and livestock production and feed demand are then used to adjust exports and human consumption to ensure market balance between production, domestic consumption and trade.

The initial outputs from all the national calculators are combined at the global level to calculate the adjustments necessary to ensure a global balance of trade, by adjusting each country’s exports until the combined global import requirements can be met. These adjustments are then incorporated into each national calculator to compute the final outputs.

Greenhouse gas emissions are calculated using standard emission factors for agricultural production, and a land-use transition matrix approach. Transitioning to a land use with a lower carbon stock results in large emissions of carbon dioxide in the conversion year, but transitioning to a higher carbon stock is assumed to sequester carbon linearly over the time taken for the new land-use type to mature. The assumptions are presented in the Supplementary Information (Table S3 and accompanying notes).

### The UK version of the FABLE calculator

The UK version of the FABLE calculator uses FAOSTAT data on land use and national commodity balances (Tables S1 and S2). For forestry, we modified the standard FABLE calculator to split the single land-use category of “forests” into semi-natural forest (mainly broadleaved) and plantation forest (mainly coniferous), which are assigned different carbon stocks and regeneration rates (Table S3) and different biodiversity values (only semi-natural forest is assumed to support biodiversity). Expansion of farmland or urban areas is not allowed in unprotected forest, in line with historic land-use patterns in the baseline period (2000–2005) and consistent with the UK Forestry Standard (Forestry Commission 2017). For afforestation, we assume that the share of new forest that can support biodiversity is the same as the proportion of existing broadleaved forest (49%).

The land-use GHG emission factors in the standard FABLE calculator were replaced by UK-specific factors that include changes to the carbon stored in soil as well as above-ground vegetation. We also included emissions or sequestration due to transitions between cropland and pasture, and loss of carbon in farmland soils from urban expansion.

The standard FABLE calculator uses the biodiversity indicator ‘land where natural processes predominate’. This indicator is not very relevant to the UK, which is a highly urbanised and agricultural landscape. Therefore, we modified this indicator to report on ‘land that can support biodiversity conservation’, which includes the land-use categories other natural land (defined as wetlands, heathland, scrub and some rough grassland; see Table S2) and semi-natural forests, but not plantations. We also include the ‘not relevant’ category (water and bare ground), which are also important for biodiversity although they do not change.

The FABLE calculator is calibrated to match historic data for the first three time steps (2000, 2005, 2010). This is achieved primarily by calculating the crop productivity by dividing historic FAO data on crop production by cropland area, and by calculating the ruminant density by dividing the FAO data on herd size by the pasture area, for those time steps. From 2015 onwards, the scenario assumptions are used to adjust the future evolution of parameters such as crop productivity or food waste using ‘shifters’. It is therefore possible for projections from 2015 to 2020 to depart from historic data. This is a limitation of the model, and in the next phase of development it will be important to update the calibration period to extend to 2020.

Further details of the UK modifications to the standard FABLE calculator are described in the ESM (‘The UK version of the FABLE calculator’).

### Pathways of UK policy ambitions

The UK pathways were co-created with 27 relevant UK stakeholders through a series of workshops and consultations in 2019–2020. Stakeholders included key experts and policymakers from relevant organisations including the Department for Environment, Food and Rural Affairs (Defra), the Department for Business, Energy and Industrial Strategy (BEIS), the Department for International Trade (DIT), the Department for Agriculture, Environment and Rural Affairs in Northern Ireland (DAERA), the Scottish Government, the Welsh Government, the UK Climate Change Committee (CCC) (an independent statutory body which provides advice to the UK government and monitors the UK’s progress towards achieving its net zero policy goal by 2050, for meeting UK climate targets), the Royal Society, the Royal Academy of Engineering, and UK Research and Innovation (UKRI). They were selected by approaching personal contacts of the FABLE project team, who were asked to identify suitable people in their organisations.

Following a stakeholder workshop in October 2019 to introduce the FABLE approach and gather feedback on key policy options that FABLE could explore, we prepared a short ‘straw man’ document containing our best estimates of the parameters needed for each pathway. These were based on key policy scenarios prepared by the CCC, specifically the Medium and High Ambition scenarios from a report on land-use policy (CCC 2018). Stakeholders were then invited to contribute to the development of the pathways by commenting (by phone or email) on this initial document, and we also requested suggestions for key policy documents or data sources that could help inform the selection of suitable parameters. A conference call was then held in February 2020 to discuss the parameterisation of the pathways, after which we circulated revised pathway parameters for further comments.

Three pathways were co-created with stakeholder input: (i) a *Current Trends* pathway that corresponds to current policies and represents a limited response to the challenge of meeting future targets; (ii) a *Sustainable Medium Ambition* pathway that corresponds to a future in which significant efforts are made to adopt sustainable policies and practices and represents a realistic yet not comprehensive response to future challenges; and (iii) a *Sustainable High Ambition* pathway that corresponds to a future in which very ambitious climate targets are achieved, in line with the UK government commitment for net zero GHG emissions by 2050, and represents a transformational switch towards more sustainable policies, at the upper limits of political, social and technical feasibility. The final pathway assumptions and related parameters are summarised in Table 2 and listed in detail in Table S5.

The pathways build on existing scenarios already used by UK policymakers, especially those developed by the CCC. Specifically, we drew on CCC analysis that suggested substantial GHG emissions reductions could be achieved by reducing beef, lamb and dairy consumption by 20–50%, improving crop yields by up to 50% and reducing food waste by 20–50% (CCC 2018). These measures would free up farmland, allowing forest cover to be increased from 13 to 19% (CCC 2018). One major addition to these existing CCC scenarios was that we adopted the EatWell diet for our Sustainable High Ambition pathway (Table S6). This is a set of guidelines on the recommended consumption of major food groups for a healthy diet, developed by Public Health England (PHE 2020). To translate the EatWell diet into specific food groups characteristic of the typical UK diet, we built on the analysis by Scarborough et al. (2016).

## Results

The following sections summarise the results of the three pathways for UK land-use change, GHG emissions from agriculture, forestry and other land use (AFOLU), biodiversity, diets/consumption, and resilience (estimated in terms of the proportion and diversity of food that is produced in the UK).

### Land-use

**Fig. 1:**
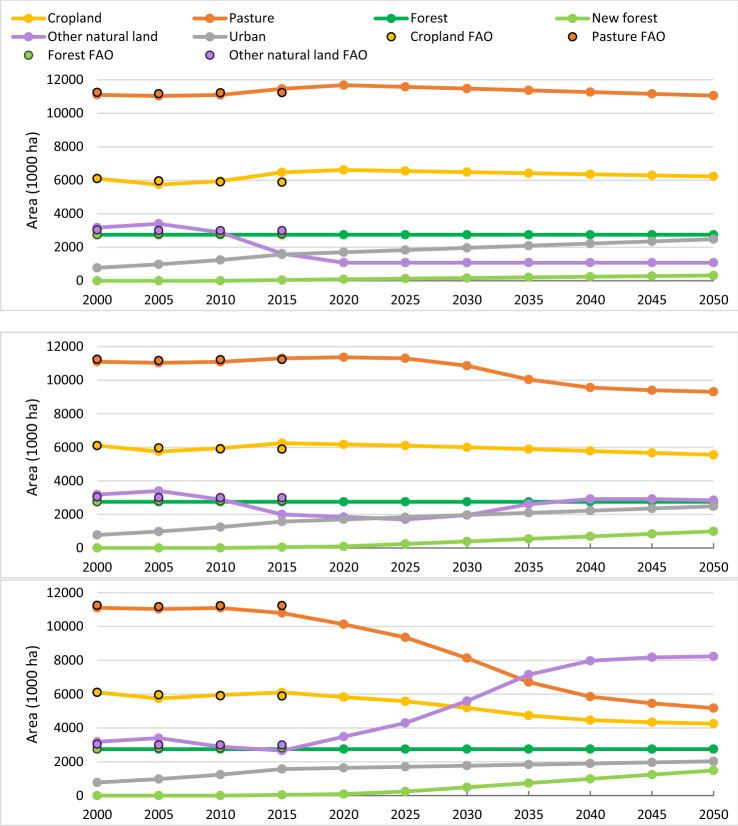
Land cover by type for the Current Trends (top), Sustainable Medium Ambition (middle) and Sustainable High Ambition (bottom) pathways. Observed data from the FAO are plotted for the first four time steps. Cumulative land-use transition matrices are presented in Table S4

**Fig. 2:**
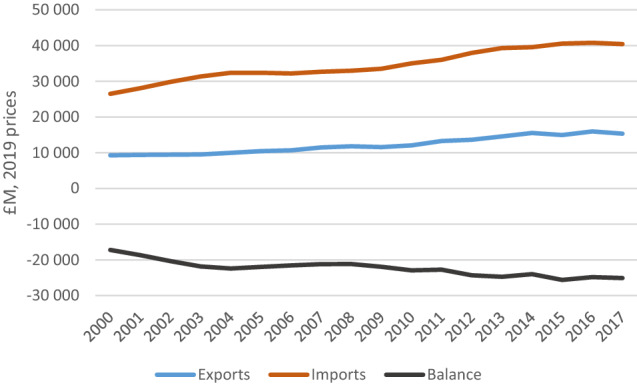
Balance of trade in food and live animals, showing long-term decline since 2000. Source: ONS (2022)

From 2000 to 2010, there is a reasonable match between the model projections and the historic land-use data from FAOSTAT. For 2015 and 2020, the model projections start to depart from the historic land-use data as they include the impacts of the scenario assumptions for the three pathways. Differences are most noticeable for the Current Trends pathway, as the ‘other natural land’ category falls below the historic value in 2015 due to projected expansion of urban, cropland and pasture area (Fig. 1). This is strongly influenced by our conservative assumption (in the absence of reliable projections) that the share of food that is imported would remain constant at 2010 levels (see Table S5). If the scenario assumptions included an increase in food imports, reflecting historic trends between 2000 and 2020 (Fig. 2), there would be less expansion of farmland in the UK and thus less loss of natural land, but there would be a corresponding expansion of farmland and loss of natural land elsewhere in the world.

Focusing on the pathways from 2020 onwards, continued expansion of urban, cropland and pasture land and contraction of natural land is projected under the Current Trends pathways until 2025, driven by population growth and increased demand for food. Pasture expansion is driven by the increase in consumption of beef, lamb and milk, and crop expansion is mainly driven by increases in demand for animal feed (barley, wheat and rapeseed), food (wheat and rapeseed oil) and biofuels (wheat). After 2025 all the unprotected ‘other natural land’ is projected to be converted to farmland, urban or new forest. This means that farmland can no longer expand to meet growing demand for food. Eventually the simulated trends in urban expansion and afforestation result in shrinkage of the area of farmland, and a decrease in consumption of domestically produced food per capita. As mentioned above, in reality it is likely that the increased demand would be met partly through increased food imports and corresponding losses in natural land in other countries, given that much of the ‘other natural land’ in the UK is low productivity.

In the Sustainable Medium Ambition pathway, the scenario assumptions concerning gradual increases in productivity, reduced food waste and decreased demand for red meat and milk consumption result in lower demand for cropland and pasture areas. From 2025 onwards, cropland and pasture area are projected to decrease, allowing ‘other natural land’ to regenerate (Fig. 1). However, after 2040, continued population growth and high rates of urban expansion reduce the amount of farmland being freed up, and eventually this causes further slight losses of ‘other natural land’.

In the Sustainable High Ambition pathway, a greater reduction in demand for agricultural land is projected, due to more ambitious assumptions about increases in productivity, reductions in food waste and a shift to a healthier, more plant-based diet (the EatWell diet). In addition, urban development is more compact in this scenario, halving the land requirement. This allows higher rates of afforestation while still freeing up land for restoration to ‘other natural land’ (Fig. 1).

### Adjustment to balance global trade

**Fig. 3:**
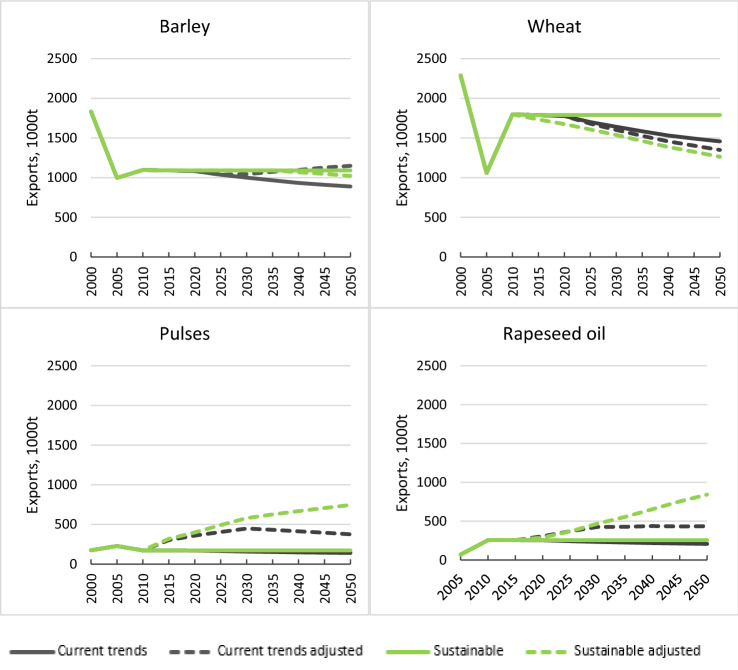
Impact of trade-balancing adjustment on FABLE projections for key UK exports

**Fig. 4:**
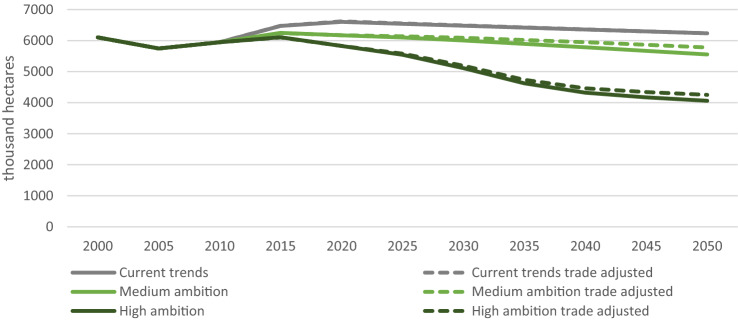
Impact of trade adjustments on cropland area for each scenario

The adjustment to balance global trade affects only the commodities for which the UK has net exports, which include barley, wheat, oats, pulses, rapeseed and rapeseed oil. Before the trade adjustment, exports in the Sustainable pathways remained constant at 2010 levels in line with our assumption of no change in export quantities (Fig. 3). However, exports were projected to decline over time for the Current Trends pathway due to constraints on further expansion of farmland. Following the trade-balancing step, exports of barley were required to increase in Current Trends, consistent with a global increase in consumption of animal feed to meet increased demand for animal products. As further expansion of UK farmland was not possible in this scenario due to lack of available land, this resulted in further reduction in the projected quantities available for consumption in the UK. In contrast, export quantities for barley decreased in the Sustainable pathways, consistent with an assumed shift to lower-meat diets in the FABLE pathways for many countries with Western style diets. For wheat, however, exports decreased in all pathways following the trade-balancing step, reflecting assumed increases in wheat exports in other FABLE countries. For pulses and rapeseed oil, the trade adjustments required exports to increase in all pathways, but more so for the Sustainable pathways, reflecting increased consumption worldwide as part of healthier diet scenarios. The net effect of the trade adjustments is that the cropland area is projected to increase slightly for the sustainable scenarios, as the increased demand for exports of rapeseed and pulses outweighs the reduced demand for exports of wheat and barley (Fig. 4).

### GHG emissions from AFOLU

**Fig. 5:**
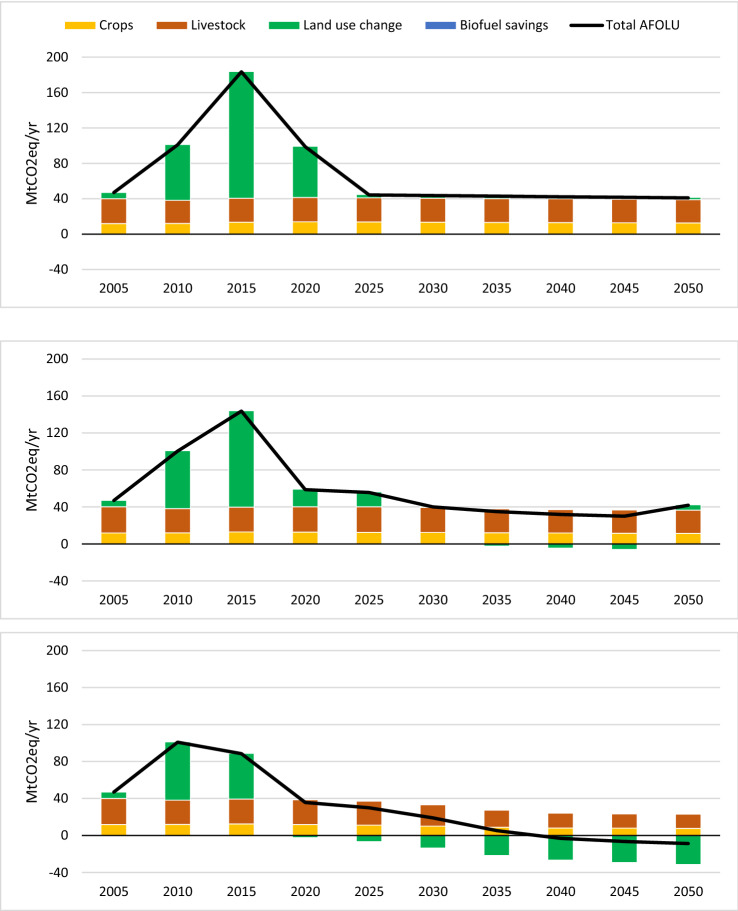
AFOLU GHG emissions by sector for the Current Trends (top), Sustainable Medium Ambition (middle) and Sustainable High Ambition (bottom) pathways. Crop emissions include N_2_O from synthetic fertilisers and crop residue, and CO_2_, CH_4_ and N_2_O from energy used during cultivation. Livestock emissions include CH_4_ and N_2_O. Land use change includes emissions when land is converted to a different type (e.g. natural land converted to farmland or urban) and carbon sequestered due to afforestation or regeneration of natural land

For the calibration period of the calculator (2000–2010), the FABLE model projects that agricultural emissions from crops and livestock remained fairly constant but with fluctuating emissions from historic land-use change (Fig. 5). These fluctuations are due to historic conversion of cropland to urban areas (2005) and then expansion of cropland onto ‘other natural land’ (2010). As the model projections for land-use start to depart from the historic land-use data in 2015 due to the scenario assumptions for the three pathways, this is reflected in the high GHG emissions simulated for 2015. This relates to the conversion of ‘other natural land’ to cropland which was greatest in the Current Trends pathway. Loss of ‘other natural land’ causes particularly high emissions because of loss of the high carbon stock in the soil (Table S3). Soil carbon is completely lost on conversion to urban areas, as all topsoil is removed, and a large proportion is lost on conversion to cropland because it oxidises when soil is ploughed. Note that in practice the soil carbon loss from land-use change would take place over several years or even decades, rather than occurring in the year of conversion, smoothing out the peaks on these charts.

**Fig. 6:**
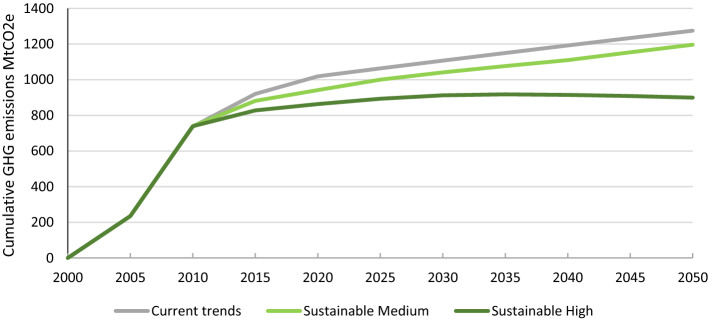
Comparison of cumulative GHG emissions since 2000 for all three pathways

Under the Current Trends pathway, this loss of natural land due to farmland and urban expansion continues until, from 2025 onwards, there is no more unprotected natural land to convert. After this, there are continued emissions from conversion of cropland and pasture to urban areas, which outweigh the small sink due to the carbon sequestration from afforestation (− 2.4 MtCO_2_e/year in 2050) and the continuing sequestration from the small amount of remaining ‘other natural land’ (− 0.9 MtCO_2_e/year). Over this period, UK agricultural emissions are projected to decrease by around 5% as farmland is converted to urban areas or afforested. In 2050, emissions from agriculture are projected to be 39 MtCO_2_e/year, of which 26 MtCO_2_e is from livestock and the remaining 13 MtCO_2_e from crops, leading to net emissions of 41 MtCO_2_e when the land-use change emissions are taken into account (Fig. 6).

From 2020 the Sustainable Medium Ambition pathway projects a decrease in the area of farmland due to dietary change, productivity improvements and reduced food waste. This enables afforestation targets to be met and allows some farmland to regenerate to natural land, providing additional carbon sequestration. However, this is almost completely cancelled out by a projected continued loss of soil carbon due to urban expansion onto farmland. This results in land use acting as only a small net sink from 2035 to 2040, and becoming a source again in 2045 as some natural land is lost to urban expansion. Emissions from crop production and livestock are projected to decrease only slightly over this period, as the emission savings from reduced consumption of beef, lamb and dairy are partly offset by emissions from the assumed increased consumption of meat from pigs and chickens. As a result, by 2050 net GHG emissions are projected to be slightly higher than the Current Trends pathway, although total GHG emissions from 2005 to 2050 are 80 MtCO_2_e lower (Fig. 6).

Under the Sustainable High Ambition pathway from 2020 onwards, a much greater decrease in emissions from cropland and livestock is projected, as farmland is freed up due to ambitious dietary change, productivity improvements and reduced food waste. Together with lower land loss to urbanisation (due to more compact development), this would allow significant regeneration of farmland to natural land, with associated increases in carbon sequestration. Combined with the higher rates of afforestation, this sequestration is projected to outweigh the emissions from agriculture leading to negative emissions from the whole AFOLU sector from 2040 onwards, reaching – 9 MtCO_2_e in 2045. Total emissions from 2005 to 2050 are projected to be 377 MtCO_2_e less than in the Current Trends pathway (Fig. 6).

### Biodiversity

Under Current Trends, protected areas are assumed to remain at the current 27.6% of total land, below the FABLE target of 30%. Also, these areas include National Parks and Areas of Outstanding Natural Beauty that include a considerable proportion (around 80%) of intensively managed farmland or plantation forestry with low biodiversity value. Under the Sustainable Medium Ambition pathway, this area increases very slightly to 27.9% of total land, based on the assumption that 0.5 Mha of land will be set aside for nature recovery, as expressed in the 25 Year Environment Plan for England and Wales (HM Government 2018), and that this area will be protected. As the FABLE calculator assumes that this land is the same mix of forest, farmland and other natural land as in currently protected areas, this means that only about 20% of the new protected area is recognised as natural land or forest. Under the Sustainable High Ambition pathway, we assume that in addition to the 0.5 Mha extra protected land for nature recovery, all currently unprotected peatland (45% of the total area of peatland) is protected from conversion to other land uses, adding a further 0.42 Mha of natural protected land and bringing the total protected area to 29.6% of total land, just under the 30% global target (Table 1). In addition, all three pathways assume no deforestation of existing woodlands, so the target for no global net deforestation is always achieved at the UK level.

**Fig. 7:**
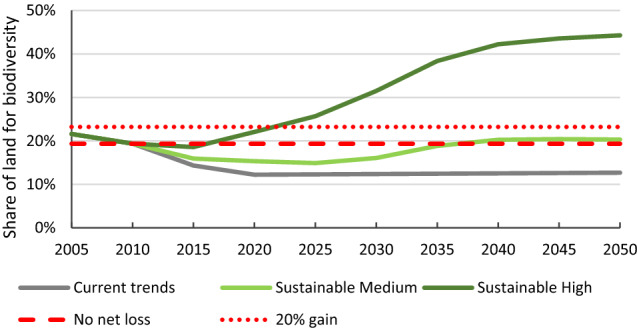
Share of land that can support biodiversity conservation under the Current Trends, Sustainable Medium Ambition and Sustainable High Ambition pathway, compared to the targets of no net loss or a 20% net gain compared to 2010

**Fig. 8:**
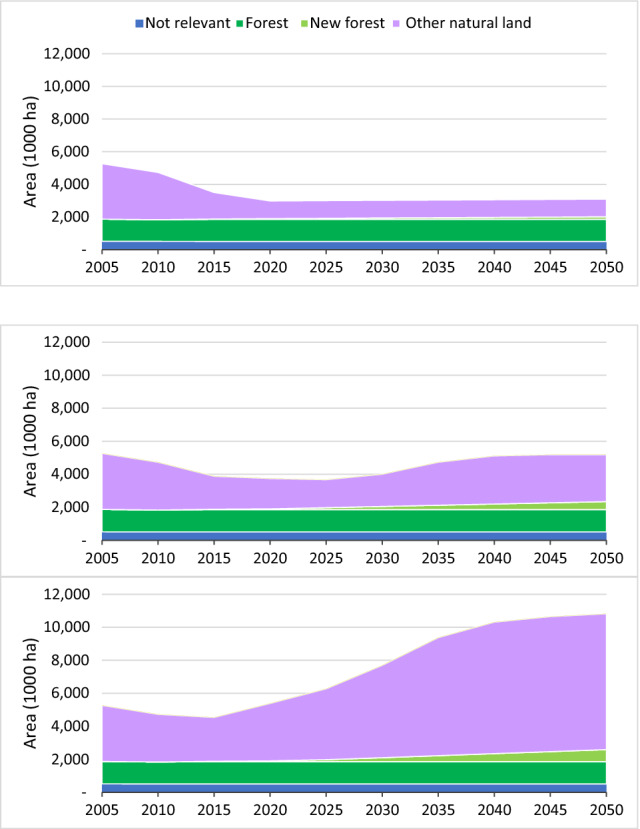
Area of land that can support biodiversity conservation for the Current Trends (top), Sustainable Medium Ambition (middle) and Sustainable High Ambition (bottom) pathways

The remaining biodiversity target is to increase the share of land that supports biodiversity conservation (semi-natural forest, other natural land and water) (Fig. 7). All pathways are dominated by the loss or regeneration of ‘other natural land’, with a smaller contribution from creation of new semi-natural woodland (Fig. 8). Under Current Trends, the share would fall from 19% in 2010 to just 12% in 2030, as all the unprotected ‘other natural land’ is converted to farmland and urban development. Beyond that, it gradually increases to 13% in 2050 due to creation of new forest, half of which is assumed to be semi-natural woodland that can support biodiversity. However, the full afforestation target cannot be met. Under the Sustainable Medium Ambition pathway the share increases slightly from 19% in 2010 to 20% in 2050 as natural land is able to regenerate and new woodlands are planted. If all the new forest was created as semi-natural native woodland managed for biodiversity, the total could increase to 23%, just meeting the target of a 20% increase from 2010 levels. In contrast, under the High Ambition Sustainability pathway both of the targets are projected to be achieved, with an increase to 44% by 2050, well above a 20% gain, or even to 47% if all the new forest was semi-natural native woodland.

### Food security and dietary change

**Fig. 9:**
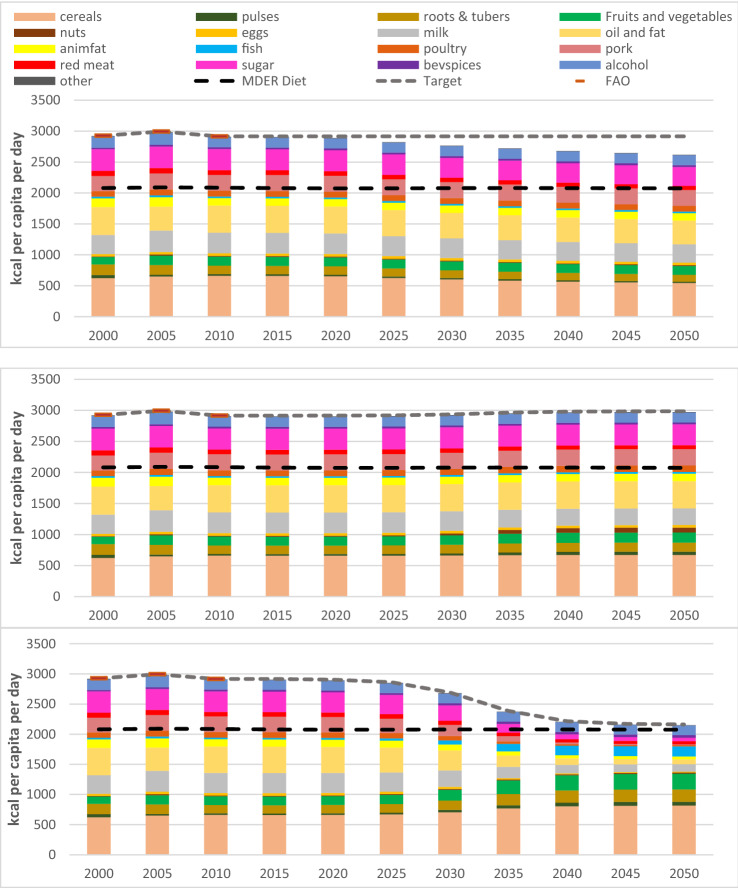
Consumption of different food groups for the Current Trends (top), Sustainable Medium Ambition (middle) and Sustainable High Ambition (bottom) pathways. Black dashed line indicates the minimum daily energy requirement. Grey dashed line indicates the target daily calorie consumption per capita based on population food demand

Under the Current Trends pathway, we assume a continuation of the current UK diet, with a target calorie consumption of 2915 cal per capita. However, when farmland can no longer expand because all unprotected natural land has been converted to other uses, and given our assumption that the share of food imports does not increase, this target calorie consumption would not be met (Fig. 9). Nevertheless, at 2618 cal per capita, the feasible consumption is still 40% higher than the minimum daily energy requirement of 2075 cal in 2050, reflecting the high level of over-consumption in the UK, which is associated with 29% of adults being obese and 63% overweight (NHS 2020). Protein consumption is projected to be 76 g/capita/day, within the recommended range of 52–182 g/capita/day (10–35% of recommended calorie consumption), but fat consumption is 120 g/capita/day, well above the recommended range of 46–69 g/capita/day (20–30% of calorie consumption).

Under the Sustainable Medium Ambition pathway, we assume that consumption of red meat (beef and lamb) and dairy produce decreases by 20%, being replaced by increased consumption of pork, poultry, nuts, pulses, fruit and vegetables. As farmland does not need to expand in this scenario, there would be no constraints on meeting the target consumption of 2985 cal/capita/day, which is slightly higher than the current UK diet and is 44% higher than the minimum daily energy requirement. There is little evidence of health benefits from this pathway because fat consumption is projected to remain at 133 g/capita/day, even higher than in Current Trends, because there are no constraints on food production.

Under the Sustainable High Ambition pathway, we assume a shift to the EatWell diet, which is the UK Government’s recommendation for a healthy diet. This diet has much lower consumption of meat, dairy, fat and sugar, and higher consumption of cereals, nuts, pulses, fish, fruit and vegetables. This would lead to a decrease in the target average daily calorie consumption per capita to 2161 cal per capita, only 4% above the minimum dietary energy requirement in 2050. Fat consumption is projected to reduce to 54 g/capita/day, within the recommended range, while protein consumption remains at 87 g/capita/day.

### Resilience of the food and land-use system

We use two indicators to assess the UK’s resilience to disruptions in food supply chains: (i) the proportion of food consumption that is produced in the UK; and (ii) the diversity of production and trade. The share of food grown in the UK has declined from a peak of 78% in 1984 (Defra 2014), a time of high CAP production subsidies, to level off at 60–64% since 2010 (Defra 2020). Out of all the major food groups, the UK only produces more than it consumes for cereals. It produces almost no beverages, spices or nuts, and produced only 45% of its fruit and vegetables in 2000, falling to 30% in 2010 (FAO 2020).

Under the Current Trends pathway, the proportion of food produced in the UK is projected to continue to decrease for the majority of agricultural products from 2010 to 2050, with the exception of cereals. Under the two sustainable pathways, slight increases in UK production are projected for most food groups, and especially for oilseed and pulses, as land for food production is freed up and demand for feed crops is reduced due to dietary change and productivity improvements.

Although the UK imports a wide range of foods, cropland is focused on producing just a few crops (wheat, barley and oilseed rape), which are also the main exports. Under the Current Trends pathway, this low level of crop diversity is projected to continue until 2050. In contrast, under the sustainable pathways, crop diversity is projected to increase slightly as less land is required to grow barley for animal feed. This could imply that the UK could be more resilient to environmental change and potential disruptions in global trade.

## Discussion

### Key findings

This study shows that the UK food and land-use system is under extreme pressure. Our analysis projects that if current trends continue, we face the prospect of complete loss of our remaining unprotected open natural land (heather moorland, bog, marsh and scrub), due to farmland expansion and urban development (and/or losses of natural land in other countries if imports increase, and/or conversion of semi-natural grassland to intensive grassland in the UK). This would release vast stores of carbon from soils and vegetation, as well as having catastrophic impacts on biodiversity. With only 13% of UK land being projected to be able to support biodiversity conservation, it would be impossible to deliver NBSAP targets for protecting and expanding the area of priority habitats and creating nature recovery networks. Even modest targets for afforestation would not be achieved due to lack of available land, further undermining UK climate targets. Given that much of the remaining natural land in the UK is low productivity moorland or bog, some or all of the increased demand is likely to be met through increased food imports, which would exacerbate environmental damage in other countries, contributing to a collective failure to meet global climate and biodiversity targets.

In contrast, the sustainable pathways show how we can meet our climate and biodiversity targets, but only through very ambitious policies to encourage a shift to healthy, plant-based diets, sustainably increase crop and livestock productivity, reduce food waste, and control urban expansion. These policies must be designed carefully to manage trade-offs and deliver multiple sustainability objectives.

Our analysis indicates that sustainable land-use policies could turn the AFOLU sector from an emission source to a sink. However, the limited savings of the Sustainable Medium Ambition pathway will not be sufficient to do this, as the 20% reduction in ruminant meat and milk consumption is partly offset by increases in feed crops to support greater production of poultry and pork. The Sustainable High Ambition pathway, however, is projected to deliver a 56% reduction in emissions compared to Current Trends. This is comparable with the CCC estimates that GHG reductions of between 35 and 80% are possible, based on conversion of 25–30% of land that is currently used for food production to other land uses and an increase from 13% forest cover to 19% (CCC 2018). As our sustainable pathways were strongly based on the CCC scenarios, this gives additional confidence in the FABLE model. Our results are also comparable with Milner et al. (2015) who showed that GHG emissions could be reduced by 17% by adjusting the UK diet to WHO recommendations or by more than 40% with more drastic dietary changes. Sun et al. (2021) have also demonstrated that shifting to the EAT-Lancet diet in high-income nations would reduce direct agricultural production emissions by 61.5% compared to 2010.

The Sustainable High Ambition pathway would enable the AFOLU sector to contribute 4% of the UK target of a 68% reduction in GHG emissions compared to 1990 (BEIS 2021), and a net sink of 9 MtCO_2_e/year by 2050. While this is an essential contribution, it also highlights that the vast majority of emission savings must still come from decarbonising the economy (c.f. CCC 2021). However, our study shows that there are many further benefits and synergies from sustainable land-use policies. In particular, dietary change is not only essential to free up land for nature recovery and carbon storage but can also deliver major health benefits through reducing over-consumption of fat and increasing intake of healthy plant-based foods. For example, Milner et al. (2015) estimated that following WHO dietary recommendations could increase average UK life expectancy by over 8 months. Multiple benefits can be maximised through community-led design and planning of restoration at landscape scales, delivering a connected network of ‘nature-based solutions’ that support climate adaptation, health and well-being as well as climate mitigation and biodiversity (Smith and Chausson 2021).

Policymakers also need to be aware of potential conflicts and trade-offs between land-use policy objectives. Afforestation policies in particular need to be carefully designed (Matthews et al. 2020). Although commercial plantations can provide sustainable timber, which can help to store carbon if used in long-lived products, they often consist of monocultures of non-native conifers with little biodiversity value (Messier et al. 2021). In addition, they can displace carbon-rich and biodiverse habitats such as semi-natural grassland, heathland or bog (Gómez-González et al. 2020). In the Current Trends pathway, despite a relatively modest amount of afforestation, all other types of natural land are projected to be lost except for that in protected areas. Similarly, Wilkes et al. (2020) showed that creating 4.2 million ha of new forest in the areas identified as suitable by Bastin et al. (2019) would result in the loss of 30–50% of the UK’s ecologically valuable habitats, 44% of improved grassland and up to 21% of protected land. In addition, planting trees on peat can lead to emissions that outweigh the carbon sequestered as the trees grow (Friggens et al. 2020; Matthews et al. 2020; Sloan et al. 2018). Although the UK Forestry Standard precludes planting on deep peat, recent studies suggest that even shallow peat should be avoided (Matthews et al. 2020). Although our scenarios did not envisage a major expansion of biofuel use in the UK, the CCC scenarios (CCC 2018) do include expansion of biofuels, and this would also involve similar caveats and trade-offs.

Current targets for expansion of housing and infrastructure will also affect our ability to meet climate and biodiversity targets, through loss of carbon stored in soil and loss, degradation and fragmentation of wildlife habitats. Our results show the benefits of shifting to more compact developments, and impacts could be further minimised by avoiding development on high quality farmland, retaining existing high-carbon habitats such as trees and hedgerows, and building in networks of new green infrastructure such as street trees, parks and green roofs (Choi et al. 2021).

A further potential trade-off involves the highly ambitious productivity improvements in our sustainable pathways. Not only is the feasibility of these improvements highly uncertain, but stakeholders emphasised that they should not rely on greater use of agro-chemicals, with adverse impacts on air and water quality (Bell et al. 2021), biodiversity and GHG emissions, or through increased livestock density leading to overgrazing, soil erosion and water pollution (e.g. Rounsevell and Reay 2009). Although a certain degree of ‘sustainable intensification’ has been ongoing since the 1990s, with decoupling of pesticide and fertiliser usage from high yields, it is unclear how much longer this trend can continue in the UK (Armstrong McKay et al. 2019). Redhead et al. (2020) found that any expansion of arable land is likely to be accompanied by widespread declines in richness of beneficial insects, even if cropping practices become less intensive. Organic farming, agroecology and agroforestry techniques with lower use of inputs can lead to increased soil carbon sequestration and benefits for the wider environment (Smith et al. 2019; FFCC 2021; Kay et al. 2019). However, if greater food imports are needed to make up for potentially lower yields this could lead to higher overseas GHG emissions (Smith et al. 2019).

Dietary change and reductions in food waste could reduce this reliance on more intensive production methods, but these options would require a fast transition of farming systems, with potential socio-economic ripple effects and impacts on farming communities. Careful policy design is therefore needed to deliver the potential synergies while mitigating or avoiding trade-offs.

### Recommendations for policymakers

To tackle these challenges and trade-offs, we recommend that:

- Existing and emerging national land-use policies within the UK nations of England, Wales, Scotland and Northern Ireland (such as the 25 Year Plan for the Environment, Environmental Land Management scheme and the National Food Strategy in England) should consider the strong evidence of the essential role that the EatWell diet and related initiatives could play in reducing GHG emissions and protecting biodiversity, as done by the Welsh Government in their recent Low Carbon Delivery plan (Welsh Government 2021, p 22). Research in the behavioural, economic and political sciences is needed to understand policy levers for dietary change.
- UK farmers, growers and producers should be supported in transitioning to a more sustainable food production system while at the same time adapting to changing dietary patterns. A shift towards more fruit and vegetable consumption could present an opportunity to expand this potentially highly profitable sector in the UK, but will require adequate policy support and a recapitalisation for these new production assets.
- Biodiversity, agriculture and forestry policies should consider the impact of large-scale afforestation on biodiversity and food production, and focus on restoring a wider range of habitats, using native species or natural regeneration where possible, adopting more sustainable management practices and aligning with nature recovery networks (Messier et al. 2021; Seddon, Smith et al. 2021).
- Housing and infrastructure development strategies should encourage more compact development patterns, avoid high value farmland, and build in a network of high quality green infrastructure. Unprotected natural land should be given more effective protection in the planning system, to safeguard carbon stores and biodiversity, including the 0.5 Mha of new nature recovery land and Nature Recovery Networks to be delivered in England.
- Major new research and investment is needed to optimise the use of agro-ecological methods that can increase productivity and soil carbon storage while minimising or even reversing adverse environmental impacts (Beillouin et al. 2022).

### Limitations and recommendations for further work

The FABLE calculator is a simplified representation of the UK food and land-use system and the analysis has certain limitations. All pasture is treated as a single category, but there are important differences in carbon storage, stocking density, productivity and biodiversity value between intensively managed and extensive (semi-natural) grassland. There are major differences between the FAOSTAT land cover data used in FABLE and the national Land Cover Map, but we were unable to use the latter as it does not yet use consistent methodology during the base period. Also, the base period needs to be extended to end in 2020 rather than 2010, so that projections match historic data better. There was also considerable uncertainty over some of the input parameters and pathway assumptions, including future trends in crop and livestock productivity, the impacts of climate change on yields (Ritchie et al. 2020), greenhouse gas emission and sequestration rates, and future livestock stocking densities. For example, although we assumed higher livestock stocking densities in our sustainable pathways, in line with the CCC scenarios, some stakeholders warned that future policies may encourage lower stocking densities in order to reduce environmental impacts such as overgrazing and water pollution. Also, the calculator does not take into account the fact that the remaining areas of natural land in the UK are typically lower productivity than the existing farmland. This means that the environmental impacts of continued growth in demand under the Current Trends scenario are a conservative estimate, as even more land would need to be converted if it was low productivity.

The GHG emission calculations from land-use change included in the calculator use a simple approach based on assumptions on the carbon content of soils and vegetation and the time for different types of land to regenerate. These are based on limited data, and the impact of peatland emissions or restoration is not yet included in the model—including the significant emissions from peatland that has been converted to farmland or forestry. This is a priority for future development of the model, as it would allow us to include the impacts of policies targeted at peatland restoration, but it is unlikely to change the main conclusions of this study regarding the differences between the pathways. This is because the majority of the emissions result from conversion of unprotected peatland (as part of ‘other natural land’) to other uses under the Current Trends scenario, resulting in very large short term emissions that far outweigh the emissions from degraded peat left in situ. For the scenarios that allow restoration of surplus farmland to natural land, as only 3% of existing farmland is on peat soil, carbon sequestration arises mainly from restoring farmland on mineral soils to a grassland–scrub–woodland–wetland habitat mosaic, with restoration to peat bog or fen where the soil type is suitable (see Table S3 notes).

It is assumed that new woodland is not planted on peaty soils, as this would give rise to additional emissions not modelled here (Friggens et al. 2020; Warner et al. 2022). However, this is an optimistic assumption as much of the low quality grazing land targeted for afforestation is on carbon-rich peaty soils. This is also a priority for future investigation.

Finally, the FABLE calculator is not spatially explicit at the sub-national level and hence does not take account of the different contexts of the four devolved nations of the UK nor spatially explicit constraints on land use. The changes in land cover (e.g. shifts from cropland to non-forest natural land) simulated in our analysis could happen anywhere in the UK. Further analysis using spatially explicit tools would be useful to identify where shifts in land cover could be prioritised to most benefit biodiversity and climate mitigation while ensuring sustainable livelihoods for farmers and affected communities.

In future work we will address some of these limitations by (i) developing versions of the FABLE calculator for the UK Devolved Administrations (England, Wales, Scotland and Northern Ireland); (ii) extending the calculator to include peatland emissions, UK-relevant bioenergy crops (coppice and miscanthus), agroforestry, hedgerow creation, and water efficiency targets; and (iii) distinguishing between improved and semi-natural grassland. We also plan to explore the spatial implications of the pathways by developing high-resolution spatially explicit models of the UK food and land-use system that can be coupled to global trade through the FABLE scenathons.

Despite these limitations, the FABLE calculator is a useful tool for exploring the part the UK can play in meeting global policy ambitions for food security, climate and biodiversity whilst taking account of trade constraints. The disruption caused by the COVID-19 pandemic and by Brexit may create new opportunities to reshape the UK food system over the next few years, and this analysis can help to inform a more holistic food and land-use strategy that responds fully to the urgent and interlinked challenges of climate change, biodiversity loss, health and sustainable development.

## Conclusions

A simple integrated assessment model, the FABLE calculator, has been used to explore potential pathways towards sustainable food and land-use systems in the UK. Our analysis shows that the simultaneous deployment of a suite of ambitious policies encompassing dietary change, productivity improvements and reductions in food waste could turn the AFOLU sector from a GHG source to a significant sink, and deliver on biodiversity targets while improving human health. However, careful policy design will be needed to deliver these multiple benefits while avoiding adverse trade-offs. In particular, policymakers need to ensure that afforestation and urban expansion do not have adverse impacts on biodiversity, carbon or food security, or shift impacts overseas through increasing imports of food. In addition, while productivity improvements are needed, they should focus on ecologically sound methods and not unsustainable intensification that damages biodiversity. Critically, the food and land-use sector will need stronger and more joined-up policy support in order to take full advantage of the synergies and manage trade-offs while transitioning to more sustainable and healthy production systems.

## Supplementary Information

Below is the link to the electronic supplementary material.

Supplementary file1 (DOCX 184 kb)
