# Supplementary material for: Sustainable pathways towards climate and biodiversity goals in the UK: the importance of managing land-use synergies and trade-offs
Source: Sustain Sci. 2022 Nov 7;18(1):521–38. doi: 10.1007/s11625-022-01242-8 (PMC9640857; doi:10.1007/s11625-022-01242-8)
Supplement: Supplementary file 1 — Supplementary file1 (DOCX 184 kb) [file 11625_2022_1242_MOESM1_ESM.docx]

Sustainable pathways towards climate and biodiversity goals in the UK:

the importance of managing land-use synergies and trade-offs

Alison C. Smith^1,*^, Paula A. Harrison^2,*^, Nicholas Leach^1^, H. Charles J. Godfray^3^, Jim W. Hall^1^, Sarah S. Jones^2,4^, Sarah Gall^1^ & Michael Obersteiner^1^

* Joint first authors; [PaulaHarrison@ceh.ac.uk](mailto:PaulaHarrison@ceh.ac.uk); [Alison.Smith@eci.ox.ac.uk](mailto:Alison.Smith@eci.ox.ac.uk)

^1^ Environmental Change Institute, University of Oxford, South Parks Road, Oxford, OX1 3TB, UK

^2^ UK Centre for Ecology & Hydrology, Library Avenue, Bailrigg, Lancaster, LA1 4AP, UK

^3^ Oxford Martin School, University of Oxford, 34 Broad St, Oxford OX1 3BD, UK

^4^ Lancaster University, Bailrigg, Lancaster, LA1 4AP, UK

**Electronic Supplementary Material**


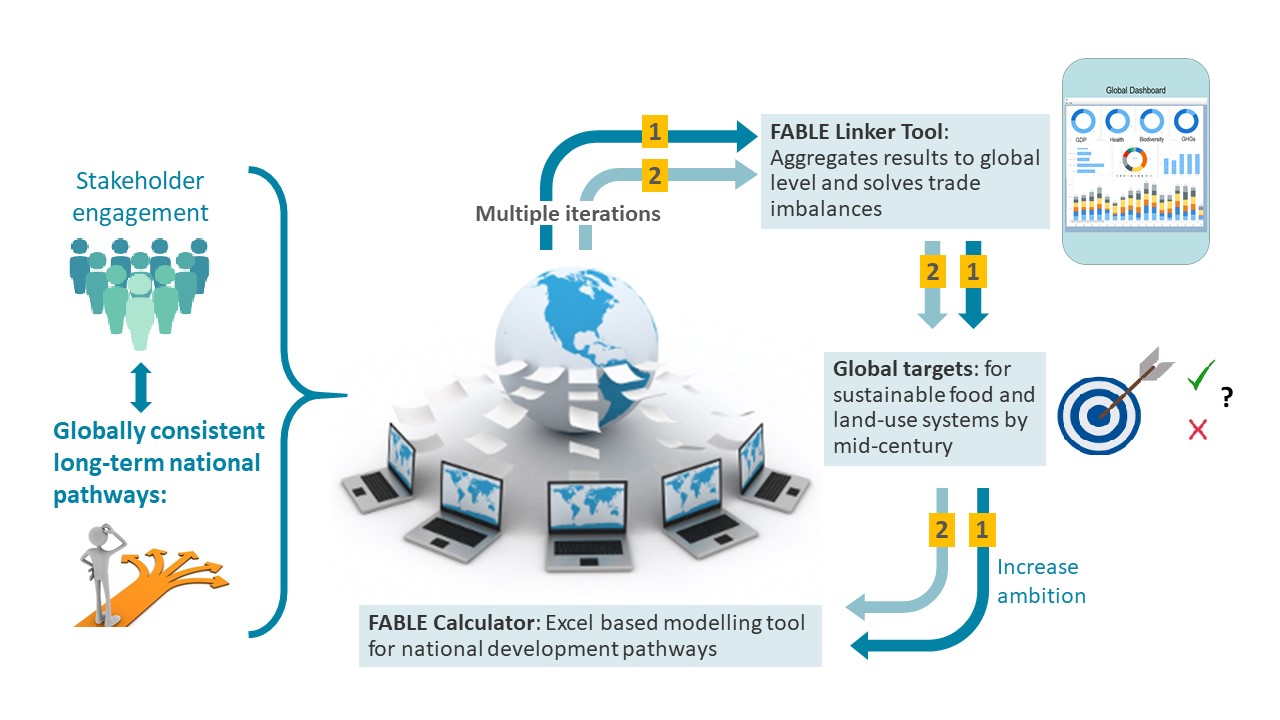


**Figure S1: Graphical summary of the iterative FABLE scenathon approach.** The 2020 FABLE scenathon consisted of two iterations and each iteration had two rounds. In the first round, trade was defined by each country based on assumptions about the evolution of its exports and imports. In the second round, national pathways were constrained by trade quantities that are consistent at the global level. At the start of each iteration, each country team worked with relevant stakeholders, such as national policy-makers, to develop pathways that are consistent with the agreed global targets. These pathways are then simulated using the FABLE Calculator, a simplified tool that enables rapid and transparent generation of pathways towards sustainable land-use and food systems using national-level data. The outcomes of the national pathways are submitted to the FABLE Linker tool, which aggregates results from the 20 country teams and seven rest of the world regions to the global level. During the second round of an iteration trade exports are corrected based on the historically reported trade imbalances for each commodity. A global dashboard enables country teams to assess their contribution to the global targets. This informs the second iteration of the scenathon, where if all or some global targets are not met, country team attempt to increase their level of ambition so that collectively they meet the global targets.

# The UK version of the FABLE Calculator

## Land-use change in the FABLE Calculator

The FABLE Calculator is designed to be used with the FAOSTAT database, as this compiles national commodity balance and land use data in a common format. However, countries are encouraged to use alternative land cover datasets if they are more accurate, and to tailor the tool to reflect national circumstances and priorities. We considered using the UK CEH Land Cover Map (Rowland et al., 2017), but this is not currently available in a consistent format due to methodology changes over the base year period (2000 to 2015), so we therefore continue to use the default FAOSTAT data (Tables S1 and S2).

**Table S1: FAO Land use data for the UK**

| Land use | 2000 | 2005 | 2010 | 2015 |
| --- | --- | --- | --- | --- |
| Forest | 2749 | 2811 | 2814 | 2815 |
| Other natural land | 3039 | 3000 | 2994 | 2992 |
| Grassland | 11244 | 11168 | 11222 | 11233 |
| Cropland | 6101 | 5960 | 5901 | 5888 |
| Urban | 775 | 981 | 990 | 994 |
| Not relevant | 521 | 510 | 508 | 508 |

Source: FAOSTAT Land Cover dataset <https://www.fao.org/faostat/en/#data/LC/metadata> which is derived from satellite data (MODIS, ESA-CCI and COPERNICUS). See <https://fenixservices.fao.org/faostat/static/documents/LC/LC_e_2021.pdf> for more detail.

**Table S2: Mapping of FAO land cover classes to FABLE land cover classes**

| FAO land cover class | FABLE land cover class |
| --- | --- |
| Artificial surfaces (including urban and associated areas) | Urban |
| Herbaceous crops | Cropland |
| Woody crops | Cropland |
| Grassland | Grassland |
| Tree-covered areas | Forest |
| Shrub-covered areas | Other natural land |
| Shrubs and/or herbaceous vegetation, aquatic or regularly flooded | Other natural land |
| Sparsely natural vegetated areas | Other natural land |
| Terrestrial barren land | Not relevant |
| Inland water bodies | Not relevant |
| Coastal water bodies and intertidal areas | Not relevant |

We modified the standard FABLE calculator to split the Forest category into semi-natural forest and plantation forest, which are assigned different carbon stocks (Table S3) and different biodiversity values. We assume that existing coniferous forests (51% of all UK forests) are mainly commercial plantations of non-native species with low biodiversity value, and broadleaved forests are mainly semi-natural woodlands that can support biodiversity. This is an approximation, as a small fraction of coniferous forest is semi-natural native pine woodland with high biodiversity value, and a small proportion of broadleaved forest is commercial plantation managed in a way that delivers low biodiversity value. For afforestation, we assume that the share of new forest that can support biodiversity is the same as the proportion of existing forest, i.e. 49%.

If farmland or urban areas need to expand, the FABLE calculator allows conversion of unprotected forest and other natural land (wetlands, heathland, scrub and some rough grassland; see Table S2). Historic rates of land-use change from 2000 to 2005 are used to determine how the urban and farmland expansion is split between forest or other natural land. According to the FAO land cover data for the UK, forest cover was roughly constant from 2000 to 2005 whereas other natural land decreased slightly. Accordingly, the model projects that all future expansion takes place on ‘other natural land’. This is in line with the UK Forestry Standard, which includes an overarching presumption against the conversion of forest land to other land-uses unless there are compelling reasons in the public interest for doing so (Forestry Commission 2017).

The FABLE calculator is calibrated to match historic data for the first three time-steps (2000, 2005, 2010). This is achieved primarily by calculating the crop productivity by dividing historic FAO data on crop production by cropland area, and by calculating the ruminant density by dividing the FAO data on herd size by the pasture area, for those timesteps. From 2015 onwards, the scenario assumptions are used to adjust the future evolution of parameters such as crop productivity or food waste using ‘shifters’. It is therefore possible for projections from 2015 to 2020 to depart from historic data. This is a limitation of the model, and in the next phase of development it will be important to update the calibration period to extend to 2020.

The cumulative land use transitions from the period 2010 to 2050 for each of the three pathways are presented in Table S4.

## GHG emissions and sequestration in the FABLE Calculator

The calculator estimates emissions from crop production, including N_2_O from synthetic fertilisers and crop residue, and CO_2_, CH_4_ and N_2_O from energy used during cultivation, using emission factors from FAOSTAT (FAO, 2020). Livestock emissions include CH_4_ from enteric fermentation in ruminants, and both CH_4_ and N_2_O from manure, based on emission factors used in the GLOBIOM model (Herrero et al., 2013). Land use change includes emissions when land is converted to a different type (e.g. natural land converted to farmland or urban) and carbon sequestered due to afforestation or regeneration of natural land.

Land use change emissions in FABLE are calculated based on estimates of carbon stock. When forest or other natural land is converted to farmland or urban, the difference in carbon stock between the two land use types is assumed to be lost immediately. However, when new forest is planted or natural land regenerates from abandoned farmland, carbon sequestration is estimated as the difference in carbon stock divided by the years taken for the forest to grow to maturity or the natural land to regenerate. The standard land use GHG emission factors in the generic FABLE calculator were replaced by UK-specific factors that include changes to the carbon stored in soil as well as above-ground vegetation, and we split forest into semi-natural and plantation, with different carbon stocks and regeneration rates (Table S3). We also modified the calculator to include emissions or sequestration due to transitions between cropland and pasture, and loss of carbon in farmland soils from urban expansion. However, we do not include emissions from loss of soil carbon during cropland cultivation. More importantly, we do not take account of emissions from degraded peatland, or from peatland that is now used for forestry or farmland. These additional emission sources are priorities for future improvements to the model.

**Table S3: GHG emission factors and assumptions regarding time taken to regenerate each type of land cove**r

| Land use | % of forest carbon stock | Years to regenerate | Tonnes C/ha | Tonnes CO_2_/ha |
| --- | --- | --- | --- | --- |
| Semi-natural forest | 100% | 110 | 329 | 1206 |
| Plantation | 50% | 60 | 164 | 603 |
| Cropland | 15% | NA | 49 | 181 |
| Pasture | 20% | 20 | 66 | 241 |
| Other natural land | 60% | 125 | 197 | 724 |
| Urban | 0% | NA | 0 | 0 |

**Notes on Table S3**.

Following the standard FABLE Calculator methodology, forest carbon stock was defined from FAO data and carbon stock for other land cover types was estimated as a proportion of forest carbon. Sequestration rates for afforestation and regeneration of natural land are then estimated by dividing the carbon stock by an estimate of the number of years it would take to regenerate the land cover.

For the UK calculator we revised the default FABLE values, making the following changes.

1. As soil carbon forms a high proportion of total carbon stock in UK ecosystems, and much of this carbon can be lost if the ecosystem is converted to urban areas or cropland, we revised all estimates to account for the total carbon stored in each land cover type, rather than only the aboveground biomass.
2. We divide forest into semi-natural forest – predominantly broadleaved forests - and commercial plantations (see Methods).
3. Semi-natural forest carbon stock is taken to be the average of the values for the years 2000 (324 tC/ha) and 2010 (334 tC/ha) in the FAO Forest Carbon dataset (FAO, 2021) including carbon in above and below ground biomass, dead wood, litter and soil. This is consistent with a major Natural England review which estimates carbon stock in 100 year old mixed native broadleaved semi-natural woodland as 354 tC/ha, for 1m soil depth, with medium-high confidence, within a range of 149 to 517 tC/ha (Gregg et al., 2021).
4. We replace the default FABLE assumption that semi-natural forest can regenerate its carbon stock in 80 years with a UK-specific assumption of 110 years, to take into account i) the relatively slow growth of UK temperate broadleaved forests, and ii) the long time needed to recover soil carbon stocks as well as above-ground carbon. The value of 110 years is consistent with the UK’s Woodland Carbon Code calculator (WCC, 2021), where carbon sequestration rates for typical broadleaved species in unmanaged forests tend to decline from an early peak to reach a low, steady value between 100 and 120 years after planting. This is consistent with an observation of soil carbon sequestration of about 0.5 tC/ha/y (1.8 tCO_2_e/ha/y) in afforestation of arable land in one site in England, which found that soil carbon (to a depth of 40cm) recovered to almost the same levels found in ancient woodland after 50–110 years (Ashwood et al., 2019).
5. Using our assumptions of 339 tC/ha and 110 years to regenerate gives an estimate of 3 tC/ha/y or 10 tCO_2_/ha/y for the sequestration rate for a new forest. For comparison, the default FABLE estimates are 63.7 tC/ha for forest carbon stock and 50 years to regenerate, giving a lower sequestration rate of 1.3 tC/ha/y or 4.7 tCO_2_/ha/y. However, the revised rate is in line with the sequestration rate estimates of 7 tCO_2_/ha/y for 100 year old mixed native broadleaf forest or 14 tCO_2_/ha/y for 30 year old mixed native broadleaf forest from Gregg et al. (2021).
6. In the UK calculator we have also modified the formula for sequestration rate to take into account the initial land cover type, so that the sequestration is calculated from the difference in carbon stock between the initial and final land covers. This is important because there will already be a certain amount of carbon in the soil of the initial land cover.
7. Parameters for plantation woodland are also based on the Woodland Carbon Code (WCC, 2021). It is assumed to have a regeneration time of 60 years, typical of rotation length in the UK, and a lower carbon stock that takes account of biomass removal during regular thinning and harvesting operations.
8. It is assumed that new woodland is not planted on peaty soils, as this would give rise to additional emissions not modelled here. However, this is an optimistic assumption as much of the low quality grazing land targeted for afforestation is on carbon-rich peaty soils. (See recommendations for further work in Discussion).
9. Cropland in England is reported to have an average carbon stock of 43 tC/ha in the top 15cm, approximately 15% of the forest stock (Gregg et al., 2021).
10. Intensively managed pasture in England is reported to have an average carbon stock of 65 tC/ha in the top 15cm, approximately 20% of the forest stock (Gregg et al., 2021). Extensive grazing land will typically have a higher carbon stock as the majority is moorland with peaty soils, though this is not modelled separately in the UK FABLE model.
11. ‘Other natural land’ comprises the FAO land cover categories of ‘Shrub-covered areas’, equivalent mainly to heather moorland (40%), ‘Shrubs and/or herbaceous vegetation, aquatic or regularly flooded’, equivalent mainly to bogs, fens, marshes and swamps (44%), and ‘Sparsely natural vegetated areas’ (16%), equivalent to sparse montane vegetation. Peat bog has extremely high soil carbon stores, entirely dependent on the depth of the peat which can be up to 6.5m, but with typical values of around 800 Mt C/ha for 2m depth (Gregg et al., 2021). Heather moorland also mostly has peaty soil, with reported carbon stocks ranging from 90 to 112 tC/ha (Gregg et al., 2021). A weighted average (assuming 30tC/ha for sparsely vegetated land and 90 tC/ha for heather moorland) implies that the carbon stock of ‘other natural land’ could be as high as 388 tC/ha, well above that of forest (329 tC/ha). Sensitivity analysis using more detailed landcover datasets results in an estimate of 332 tC/ha. However, because not all this carbon would be lost in the year of conversion (as modelled in FABLE) we use a more conservative estimate of carbon stock that is 60% that of forest (double the estimate of 30% in the generic FABLE Calculator). This is likely to overestimate emissions in the short term but underestimate them in the long term. See the Discussion for the limitations of these assumptions and the priorities for further development of the model to address these issues.
12. The generic FABLE Calculator assumes that abandoned farmland regenerates to ‘other natural land’ but it assigns this to have the same carbon stock as existing forest (rather than existing ‘other natural land’), and assumes that this regeneration takes 80 years (equivalent to a sequestration rate of 0.8 tC/ha/y using generic estimates for forest carbon stock). In the UK model, as our targets for tree planting are reflected in the creation of ‘New forest’, we assume that any surplus farmland can be restored to other types of natural land (mainly non-forest). However, it will not be possible for existing farmland to regenerate to the same mix of habitats as in existing ‘other natural land’, with a large proportion of peat bog, because only 3% of existing intensive farmland is on peat soil (estimate based on farmed peat areas from Evans et al., 2017 and total farmed areas from Rowland et al., 2017). We assume instead that abandoned farmland regenerates to a different type of ‘other natural land’ comprising a mosaic of semi-natural grassland, heath, scrub, woodland and wetland, mainly on mineral soil but including restored peat bogs in areas with peaty soils. To simulate this within the existing protocol of the FABLE model, we estimate that regenerated ‘other natural land’ will contain 60% of the forest carbon stock, and we select a regeneration time of 125 years. This is chosen to result in a realistic sequestration rate of 1.58 tC/ha/y, intermediate between estimates for reversion of arable land to grassland (0.43 tC/ha/y; Gregg et al., 2021) and growth of semi-natural woodland (3 tC/ha/y; see point 5 above). For the 3% of farmland being restored to peat bog, it could take 40 years for sequestration to begin, and it is then typically less than 1 tC/ha/y (Gregg et al., 2021). However, restoration will also reduce the emissions from degraded peat, which could be as high as 8-10 tC/ha/y for intensive grassland or cropland on peat soil (Evans et al., 2017). This is not yet included in the FABLE model but is a priority for further work.

## Biodiversity in the FABLE Calculator

The calculator reports on four biodiversity indicators: net deforestation; share of land that is protected; share of land that can support biodiversity conservation (forest and other natural land); and share of ‘land where natural processes predominate’. The last of these includes forest, other natural land, historic grassland (existing by 2010 but not created after that) and areas classified as ‘not relevant’ (water and bare ground) that fall within one of three zones: ‘low impact areas’ (far from human disturbance such as roads, cropland and urban areas; Jacobson et al (2019), ‘key biodiversity areas’ (important bird areas, Birdlife International 2019) and ‘intact forest landscapes’ (Potapov et al., 2008). However, there are very few of these areas in the UK, which is a highly urbanised and agricultural landscape, and we wanted to reflect the value of restoring land for nature even close to urban areas and intensive farmland. Therefore we report on ‘land that can support biodiversity conservation’, and we modified this indicator to include semi-natural forests but not plantations. We also include the ‘not relevant’ category (water and bare ground), which are also important for biodiversity although they do not change.

**Table S4. Cumulative land use transitions from the period 2010 to 2050 for each of the three pathways (thousand hectares).**

|  | To cropland | To pasture | To urban | To new forest | To other natural land | Total |
| --- | --- | --- | --- | --- | --- | --- |
| Current Trends | | | | | | |
| Forest | - | - | - | - | - | - |
| Other natural land | 699 | 637 | 461 | 13 | - | **1,810** |
| Cropland | - | - | 256 | 94 | 0 | **350** |
| Pasture | - | - | 394 | 174 | 0 | **568** |
| Total | **699** | **637** | **1,111** | **281** | **0** |  |
| Sustainable Medium Ambition | | | | | | |
| Forest | - | - | - | - | - | **-** |
| Other natural land | 315 | 289 | 721 | 125 | - | **1,450** |
| Cropland | - | 52 | 14 | 251 | 79 | **396** |
| Pasture | - | - | 376 | 463 | 1,197 | **2,036** |
| Total | **315** | **340** | **1,111** | **840** | **1,276** |  |
| Sustainable High Ambition | | | | | | |
| Forest | - | - | - | - | - | **-** |
| Other natural land | - | - | 331 | 185 | - | **516** |
| Cropland | - | - | 87 | 371 | 1,417 | **1,875** |
| Pasture | 268 | - | 303 | 684 | 4,384 | **5,639** |
| Total | **268** | **-** | **721** | **1,240** | **5,801** |  |

**Table S5: Detailed assumptions, parameterisation and underlying rationale for the three UK pathways.** Adapted from Smith et al. (2020).

| **Current Trends Pathway** | **Sustainable Medium Ambition Pathway** | **Sustainable High Ambition Pathway** |
| --- | --- | --- |
| **Population:** Population projection (million inhabitants) | | |
| The population is expected to reach 75.4 million by 2050 (UN medium projections). *Based on UN DESA (2017).*  (UN Medium Projection scenario selected) | | |
| **Land** | | |
| **Constraints on agricultural expansion** | | |
| We assume that there will be no constraint on the expansion of agricultural land beyond existing protected areas and under the total land boundary. *Based on lack of any UK policy to constrain expansion.* | | |
| **Afforestation or reforestation target (1,000 ha)** | | |
| We assume total afforested/ reforested area to reach 326Mha by 2050. *Based on continuation of average rate of tree planting 2014-2016, i.e. 9,000ha/y (CCC, 2018).* | We assume total afforested/ reforested area to reach 990Mha by 2050. *Based on CCC medium ambition scenario, i.e. 30,000ha/y (CCC, 2018).* | We assume total afforested/ reforested area to reach 1490Mha by 2050. *Based on CCC high ambition scenario, i.e. 50,000ha/y (CCC, 2018).* |
| **Urban expansion** | | |
| Increase of urban area from 7% of UK land area in 2015 (1.6Mha) to 10.2% (2.5Mha) by 2050, at a rate of 26,000ha/y. *Based on government projections for future housing needs (CCC, 2018; MHCLG, 2019).* | As for Current Trends. | Increase of urban area from 7% of UK land area in 2015 (1.6Mha) to 8.3% (2.0Mha) by 2050, at a rate of 13,000ha/y. *Based on assumption that land take could be half of Current Trends, e.g. if developments were more compact, following the approach used in (Thomson et al., 2018).* |
| **Biodiversity:** Protected areas (% of total land) | | |
| Protected areas remain stable: by 2050 they represent 27.6% of total land. *Based on (WDPA, 2020).* Includes National Parks, AONBs, local and national nature reserves, SSSIs, Ramsar sites and Natura sites (SACs and SPAs). Within these areas, the FABLE Calculator distinguishes between forests and other natural land, which are assumed to be unavailable for expansion of food production, and farmland, where production can continue. | Protected areas increase: by 2050 they represent 27.9% of total land. *Based on assumption that 0.5Mha of land will be set aside for nature recovery, as expressed in 25 Year Environment Plan for England (HM Government, 2018), and that this area will be protected.* Note that the FABLE Calculator currently assumes that this land is the same mix of forest, farmland and other natural land as in currently protected areas. This means that only about 20% of the new protected area is recognised as natural or forest. | Protected areas increase: by 2050 they represent 29.6% of total land. *Based on assumption that in addition to the 0.5Mha extra protected land for nature recovery, all unprotected peatland is protected, adding a further 0.42Mha of natural protected land (calculations by UK FABLE team).* |
| **Production** | | |
| **Crop productivity for the key crops in the country (in t/ha)** | | |
| In 2050, crop productivity remains at:   - 7.7 tons per ha for wheat (7.1 with climate change impacts). - 5.7 tons per ha for barley. - 43.9 tons per ha for potatoes.   *Based on FAOSTAT historic yields for 2010.* | By 2050, crop productivity reaches:   - 10.7 tons per ha for wheat (10.1 with climate change impacts). - 7.9 tons per ha for barley. - 61 tons per ha for potatoes.   *Based on assumption that yields for all crops increase by 39% from the 2010 value, in line with the revised CCC medium projection (CCC, personal communication, 2020).* | By 2050, crop productivity reaches:   - 12.7 tons per ha for wheat (12.0 with climate change impacts). - 9.4 tons per ha for barley. - 72.4 tons per ha for potato.   *Based on assumption that yields for all crops increase by 65% (in line with the revised CCC high projection (CCC, personal communication, 2020).* |
| **Livestock productivity for the key livestock products in the country (in kg/head of animal unit)** | | |
| By 2050, livestock productivity (annual production / average herd size, not carcass weight) reaches:   - 7,971 kg per head for milk. - 85 kg per head for cattle meat. - 13.7 kg per head for chicken meat.   *Based on assumption that milk yield increases by 18%, half the current rate, while other yields remain at 2015 levels, using UK agriculture statistics (Defra, 2019).* | By 2050, livestock productivity reaches:   - 7,971 kg per head for milk. - 85 kg per head for cattle meat. - 15.3 kg per head for chicken meat.   *Based on assumption that milk yield increases by 18%, half the current rate; cattle remains the same as productivity is assumed to increase via changes to stocking density; poultry assumed to increase proportional to the assumed increases in stocking density for cattle.* | By 2050, livestock productivity reaches:   - 8,669 kg per head for milk. - 85 kg per head for cattle meat. - 15.3 kg per head for chicken meat.   *Based on increase of 27% for milk yield, 75% of the current rate of increase; no further increase for cattle or chicken, to reflect animal welfare and physiology constraints/ limits to further yield increases.* |
| **Pasture stocking rate (in animal units/ha pasture)** | | |
| By 2050, the average ruminant livestock stocking density is 1.1 TLU/ha. *Based on assumption that the stocking density remains unchanged from the value in 2010 according to FAOSTAT (herd numbers divided by pasture area).* | By 2050, the average ruminant livestock stocking density is 1.2 TLU/ha. *Based on increase of 10% from 2015, the same % increase as in CEH Rothamsted high ambition scenario (Thomson et al., 2018).* | By 2050, the average ruminant livestock stocking density is 1.7 TLU/ha. *Based on increase of 50% from 2015, the same % increase as in CCC high ambition scenario (CCC, 2018), and guidance on potential densities (AHDB, 2016).* |
| **Post-harvest losses** | | |
| By 2050, the share of production and imports lost during storage and transportation is 1% for crop products and unknown (assumed zero) for livestock products. *Based on FAOSTAT data and assumption of no change from present day, but data is patchy.* | By 2050, the share of production and imports lost during storage and transportation is 0.5%. *Based on assumption of a 50% reduction in losses compared to 2015, i.e. achieving the SDG 12.3 target to halve consumer and retail waste but by 2050 rather than 2030 (WRAP, 2020).* | By 2030, the share of production and imports lost during storage and transportation is 0.5%, i.e. the target is achieved earlier than the Medium Ambition Sustainable scenario. *Based on assumption of a 50% reduction in losses compared to 2015 in line with the Courtauld 2025 Commitment (reduction of 20% across supply chain between 2015-2025) and SDG 12.3 target (halve consumer and retail waste by 2030) (WRAP, 2020).* |
| **Trade** | | |
| **Share of consumption which is imported for key imported products (%)** | | |
| By 2050, the share of total consumption which is imported remains at the 2015 values:   - 53% for other vegetables. - 88% by 2050 for apples. - 25% by 2050 for beef.   *Based on stakeholder discussions and agreement that the outcome of Brexit trade negotiations and the design of the replacement agricultural support scheme were too uncertain to allow meaningful projections of future change (see also Centre for Rural Economy and Land Use, 2018).* | | |
| **Evolution of exports for key exported products (1,000 tons)** | | |
| By 2050, the volume of exports remains at the 2015 values:   - 1.8Mt by 2050 for wheat. - 1.1Mt by 2050 for barley. - 0.3Mt by 2050 for rapeseed oil.   *Based on stakeholder discussions and agreement that the outcome of Brexit trade negotiations and the design of the replacement agricultural support scheme were too uncertain to allow meaningful projections of future change.* | | |
| **Food** | | |
| **Average dietary composition (daily kcal per commodity group) (see also Table S6)** | | |
| By 2030, the average target daily calorie consumption per capita is 2,983 kcal and is:   - 168 kcal for fruit and vegetables. - 83 kcal for ruminant meat. - 119 kcal for animal fats.   *Based on assumption of no change in current diet as in FAOSTAT.* | By 2030, the average target daily calorie consumption per capita is 2,894 kcal and is:   - 167 kcal for fruit and vegetables. - 78 kcal for ruminant meat. - 119 kcal for animal fats.   *Based on CCC medium ambition scenario (20% reduction in red meat and milk consumption by 2050, replaced with increased consumption of pork, poultry, fish, eggs, pulses and nuts) (CCC, 2018).* | By 2030, the average target daily calorie consumption per capita is 2,739 kcal and is:   - 196 kcal for fruit and vegetables. - 75 kcal for ruminant meat. - 98 kcal for animal fats.   *Based on meeting the Eatwell diet recommendations by 2050 (PHE, 2020; Scarborough et al., 2016).* |
| **Share of food consumption which is wasted at household level (%)** | | |
| By 2030, the share of final household consumption which is wasted at the household level is 14%. *Based on assumption of no change from current levels (WRAP, 2020).* | By 2030, the share of final household consumption which is wasted at the household level is 12.5%. *Based on CCC medium ambition scenario in which the share which is wasted decreases linearly by 20% from 2010 to 2050, from 14% to 11% (CCC, 2018).* | By 2030, the share of final household consumption which is wasted at the household level is 7%. *Based on Courtauld 2025 Commitment (reduction of 20% across supply chain between 2015-2025) and SDG 12.3 target (halve consumer and retail waste by 2030 (WRAP, 2020).* |
| **Biofuels:** Targets on biofuel and/or other bioenergy use (precise unit) | | |
| By 2050, biofuel production accounts for:   - 226kt of corn production. - 909kt of wheat production.   *Based on OECD Aglink projections until 2028; stable afterwards.* In future this could be modified to reflect UK policy to use coppice wood and miscanthus grass rather than food crops. | By 2050, biofuel production accounts for:   - 231kt of corn production. - 1143kt of wheat production.   *Based on OECD Aglink projections until 2028; stable afterwards.* | By 2050, biofuel production accounts for:   - 215kt of corn production. - 1053kt of wheat production.   *Based on OECD Aglink projections until 2028; stable afterwards.* |
| **Water:** Evolution of irrigation water use efficiency | | |
| By 2050, average blue water use for irrigation per ton produced is the same as in 2010. *Based on assumption of no change as there was no available data on future trends.* | | |
| **Climate change:** Crop model and climate change scenario | | |
| By 2100, global GHG concentration leads to a radiative forcing level of 6 W/m2 (RCP 6.0). Impacts of climate change on crop yields are computed by the crop model GEPIC using climate projections from the climate model HadGEM2-E without CO_2_ fertilization effect. | By 2100, global GHG concentration leads to a radiative forcing level of 2.6 W/m2 (RCP 2.6). Impacts of climate change on crop yields are computed by the crop model GEPIC using climate projections from the climate model HadGEM2-E without CO_2_ fertilization effect. | By 2100, global GHG concentration leads to a radiative forcing level of 2.6 W/m2 (RCP 2.6). Impacts of climate change on crop yields are computed by the crop model GEPIC using climate projections from the climate model HadGEM2-E without CO_2_ fertilization effect. |

**Table S6: Dietary composition of the three pathways (kcal / capita / day)**

| Product group | Current Trends (no change) | Sustainable Medium Ambition (CCC targets: 20% reduction in beef, lamb and dairy) | Sustainable High Ambition (EatWell diet) |
| --- | --- | --- | --- |
| Cereals | 660 | 672 | 817 |
| Pulses | 26 | 52 | 64 |
| Root vegetables | 136 | 147 | 206 |
| Fruit and vegetables | 148 | 166 | 264 |
| Nuts | 13 | 79 | 18 |
| Oils and oilseeds | 439 | 440 | 82 |
| Animal fat (butter) | 119 | 119 | 48 |
| Milk | 334 | 267 | 126 |
| Eggs | 42 | 41 | 15 |
| Fish | 27 | 30 | 166 |
| Poultry | 93 | 104 | 19 |
| Pork | 258 | 264 | 13 |
| Ruminant meat (beef and lamb) | 79 | 66 | 58 |
| Sugar | 344 | 341 | 59 |
| Beverages and spices | 30 | 30 | 44 |
| Alcohol | 161 | 161 | 161 |
| Other | 7 | 7 | 3 |

# References

AHDB (2016). *Planning grazing strategies for better returns*. Retrieved from Agriculture and Horticulture Development Board, UK website: <https://ahdb.org.uk/knowledge-library/planning-grazing-strategies-for-better-returns>

Ashwood, F., Watts, K., Park, K., Fuentes-Montemayor, E., Benham, S., & Vanguelova, E. I. (2019). Woodland restoration on agricultural land: Long-term impacts on soil quality. *Restoration Ecology*, *27*(6), 1381–1392. <https://doi.org/10.1111/rec.13003>.

BirdLife International (2019) Digital boundaries of Important Bird and Biodiversity Areas from the World Database of Key Biodiversity Areas. February 2018 Version.

CCC (2018). *Land use: Reducing emissions and preparing for climate change*. Retrieved from Committee on Climate Change website: <https://www.theccc.org.uk/publication/land-use-reducing-emissions-and-preparing-for-climate-change/>

Centre for Rural Economy and Land Use. (2018). *Brexit: how will UK agriculture fare when we leave the EU?* (Issue 7).

Defra (2019). *Agriculture in the United Kingdom 2018*. Retrieved from UK Department of Environment, Food and Rural Affairs website: <https://www.gov.uk/government/statistics/agriculture-in-the-united-kingdom-2018>

Evans, C., Artz, R., Moxley, J., Smyth, M-A., Taylor, E., Archer, N., Burden, A., Williamson, J., Donnelly, D., Thomson, A., Buys, G., Malcolm, H., Wilson, D., Renou-Wilson, F., Potts J. (2017). Implementation of an emission inventory for UK peatlands. Report to the Department for Business, Energy and Industrial Strategy, Centre for Ecology and Hydrology, Bangor.88pp.

FAO (2020). *FAOSTAT* [Database]. Retrieved from <http://www.fao.org/faostat/en/#home>

FAO (2021) Forest Carbon dataset, <https://fra-data.fao.org/GBR/fra2020/carbonStock/>

Forestry Commision (2017). The UK Forestry Standard: The government's approach to sustainable forestry. Forestry Commission, Fourth Edition, Forestry Commission, Edinburgh, ISBN: 978-0-85538-999-4, <https://assets.publishing.service.gov.uk/government/uploads/system/uploads/attachment_data/file/687147/The_UK_Forestry_Standard.pdf>

Gregg, R., Elias, J.L., Alonso, I., Crosher, I.E., Muto, P. and Morecroft, M. D. (2021) Carbon storage and sequestration by habitat: a review of the evidence (second edition) Natural England Research Report NERR094. Natural England, York.

Herrero, M., Havlík, P., Valin, H., Notenbaert, A., Rufino, M. C., Thornton, P. K., … Obersteiner, M. (2013). Biomass use, production, feed efficiencies, and greenhouse gas emissions from global livestock systems. Proceedings of the National Academy of Sciences , 110 (52), 20888–20893.

HM Government (2018). *A Green Future: Our 25 Year Plan to Improve the Environment*. Retrieved from UK Government website: <https://assets.publishing.service.gov.uk/government/uploads/system/uploads/attachment_data/file/693158/25-year-environment-plan.pdf>

Jacobson, A.P., Riggio, J., Tait, A.M., Baillie, J.E.M. (2019) Global areas of low human impact (‘Low Impact Areas’) and fragmentation of the natural world. *Sci. Rep.* **9**, 1–13).

MHCLG (2019). Live tables on land use change statistics: Land Use Change Statistics (LUCS) 2017-18. Retrieved May 24, 2020, from GOV.UK website: <https://www.gov.uk/government/statistical-data-sets/live-tables-on-land-use-change-statistics>

PHE (2020). The Eatwell Guide. Retrieved May 28, 2020, from GOV.UK website: <https://www.gov.uk/government/publications/the-eatwell-guide>

Potapov, P. et al. (2008) Mapping the world’s intact forest landscapes by remote sensing. *Ecol. Soc.* **13**.

Rowland, C.S.; Morton, R.D.; Carrasco, L.; McShane, G.; O'Neil, A.W.; Wood, C.M. (2017). *Land Cover Map 2015* (vector, GB). NERC Environmental Information Data Centre. (Dataset). <https://doi.org/10.5285/6c6c9203-7333-4d96-88ab-78925e7a4e73>

Scarborough, P., Kaur, A., Cobiac, L., Owens, P., Parlesak, A., Sweeney, K., & Rayner, M. (2016). Eatwell Guide: Modelling the dietary and cost implications of incorporating new sugar and fibre guidelines. *BMJ Open*, *6*(12), e013182. https://doi.org/10.1136/bmjopen-2016-013182

Thomson, A., Misselbrook, T., Moxley, J., Evans, C., Malcolm, H., Whitaker, J., & Reinsch, S. (2018). *Quantifying the impact of future land use scenarios to 2050 and beyond—Final Report* (Final Report for the Committee on Climate Change No. IT/KB 0917; p. 78). Retrieved from CEH and Rothamsted Research website: https://www.theccc.org.uk/wp-content/uploads/2018/11/Quantifying-the-impact-of-future-land-use-scenarios-to-2050-and-beyond-Full-Report.pdf

UN DESA (2017). *World Population Prospects: The 2017 Revision, Key Findings and Advance Tables* [Working Paper]. Retrieved from United Nations website:<https://esa.un.org/unpd/wpp/Publications/Files/WPP2017_KeyFindings.pdf>

WCC (2021) Woodland Carbon Code (WCC) Carbon Calculation Spreadsheet V2.4. https://www.woodlandcarboncode.org.uk/images/Spreadsheets/WCC_CarbonCalculationSpreadsheet_Version2.4_March2021_bsml.xlsb.

WDPA (2020). Protected Planet: World database on protected areas. Retrieved May 23, 2020, from https://www.protectedplanet.net/

WRAP (2020). *Food surplus and waste in the UK: Key Facts. Updated Jan 2020.* Retrieved from Waste and Resources Action Plan website: https://wrap.org.uk/sites/files/wrap/Food_%20surplus_and_waste_in_the_UK_key_facts_Jan_2020.pdf
